# *“It’s Everyone’s Problem”:* Institutionalising Multisectoral Action for Maternal Health in Meghalaya, India

**DOI:** 10.5334/aogh.4587

**Published:** 2025-03-03

**Authors:** Sapna Desai, Sharmada Sivaram, S Ramkumar, Patricia Dohtdong, Ankit Nanda, Sowmya Ramesh, Sampath Kumar

**Affiliations:** 1 Population Council Institute, New Delhi, India; 2 Government of Meghalaya, India

**Keywords:** Maternal health, Multisectoral, Implementation research, State capabilities

## Abstract

*Background:* There is widespread agreement on the potential of multisectoral action to address the social determinants of maternal health. We conducted an implementation research study in Meghalaya, a northeastern Indian state with a high burden of maternal mortality where the government initiated “Rescue Mission” to strengthen the health system and to address underlying determinants to improve maternal health indicators. The initiative was grounded in building state capability and decentralised leadership.

*Objective:* We developed a theory of change and examined implementation barriers and enablers through an implementation research study with government and community actors and institutions.

*Methods:* We conducted multiple rounds of qualitative data collection over a period of eighteen months across six districts. Participants included primary care providers in the public health system and frontline workers in thirty sampled facilities. We also interviewed officials across three government departments, observed meetings and met regularly in a feedback loop with government. Data were analysed thematically and synthesised according to pathways of change.

*Findings:* The state institutionalised multisectoral collaboration across governance levels through building technical and adaptive leadership. Processes included joint meetings at the facility, district and state levels to develop action plans and facilitate collaboration, community engagement through frontline workers and decentralised use of data. Strength of participation by different sectors varied widely; non‑health cadres reported challenges such as being accountable to multiple departments. Political priority and administrative leadership were the key elements of the State’s ability to implement a multisectoral approach. Overall, health outcomes improved and the State largely achieved its commitment to building technical skills, but also recognised the need for further investments to develop a sense of purpose amongst government officials.

*Conclusions:* Meghalaya’s experience in multisectoral collaboration demonstrates the potential of health systems reform grounded in a state capabilities enhancement approach, with a focus on participation and building decentralised leadership.

## Background

As the world moves towards reducing maternal mortality, many countries face persistent challenges related to underlying determinants of poor health and access to services [1]. In India, the government has achieved significant reductions in the maternal mortality ratio (MMR), from 556 per 100,000 live births in 1990 to 97 per 100,000 live births in 2021, higher than the rate of global decline [2, 3]. Increased access to quality obstetric services, decreasing fertility rates and strengthened health systems are some of the key factors that addressed direct causes of mortality [4, 5]. However, inequities in progress, both across regions and within social groups, call for further focus on the underlying social and structural determinants of maternal health such as socioeconomic status, gender dynamics and broader infrastructural and environmental factors [6–8].

Addressing causes of maternal mortality not directly related to health requires a multisectoral response. Wide variation in health outcomes across settings in India calls for tailored responses that account for diversity in demographic, health system, infrastructure and socioeconomic environments [9]. In India’s federal structure, health is a State subject – which allows for localised responses that engage different actors as required. While there is extensive documentation of State health system responses to maternal health, there has been relatively less analysis of multisectoral responses across government departments [5].

This paper examines the Government of Meghalaya’s multisectoral response to maternal health through building state capability and promoting decentralised leadership. In a departure from primarily supply‑ or demand‑driven maternal health interventions, the state government implemented a health systems strengthening programme that aimed to change how problems are solved, to empower decision‑makers to address local issues with locally developed, multisectoral solutions that reach the most vulnerable [10, 11]. We conducted an embedded, implementation research study to examine processes to inform Government actions as well as to identify principles towards future transferability.

### Setting

Meghalaya is a state in the northeastern region of India, with a population of over three million (Census 2011) [12]. Over 80% of its population resides in rural areas, with three main tribal communities: Khasi, Jaintia and Garos [12]. The state’s geography is characterized by hills, highland plateaus, deep valleys and a vast forest cover making several areas hard to reach and difficult to traverse. While Meghalaya has achieved success in addressing infectious diseases, maternal mortality has remained persistently higher (197/100,000 live births) than the national average [13]. Uptake of antenatal care (ANC) services and institutional deliveries is low, at a little over 50% [14].

**Figure 1:**
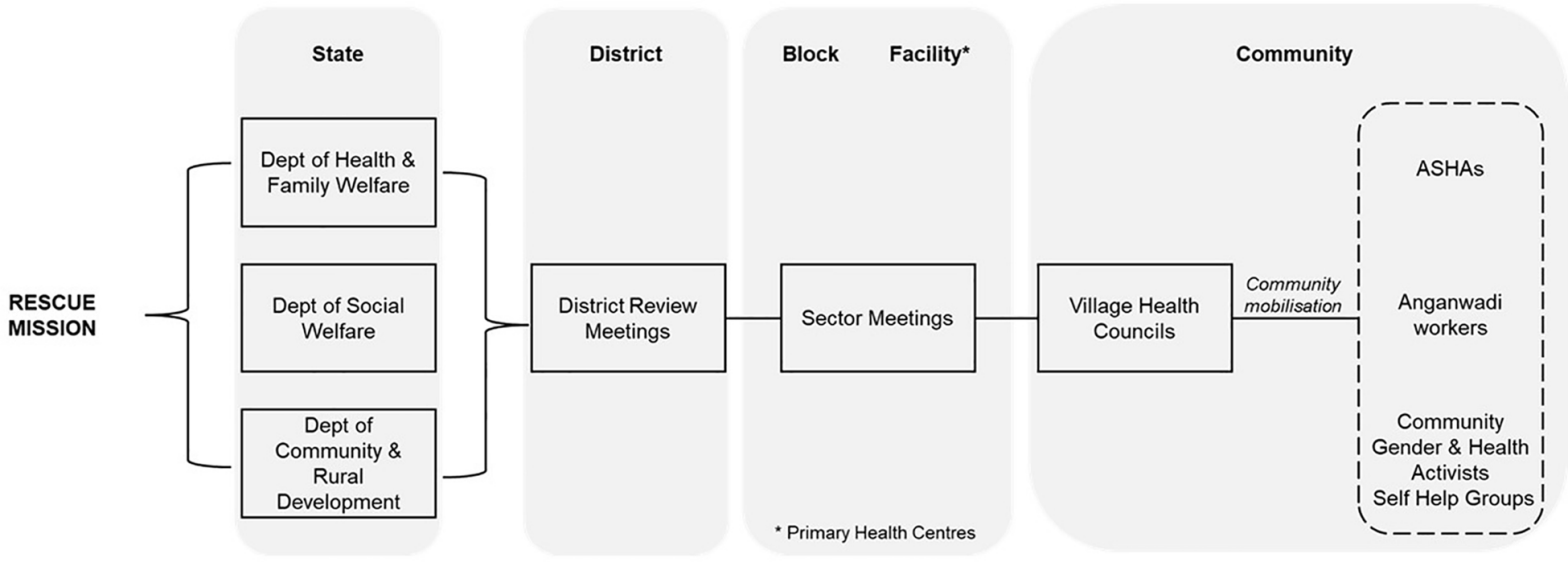
Rescue Mission Overview.

In response, the Government of Meghalaya introduced Rescue Mission, a multisectoral approach that aims to enhance state capabilities to address maternal health (Figure 1). The state government first mapped out challenges in an iterative and adaptive approach. Health systems issues included deficient infrastructure, lack of trained human resources and lack of trust in health services. Transportation in difficult terrain is limited, which hinders access for women in labour to facilities within 30 minutes. Additional underlying challenges include limited birth spacing, teenage pregnancy, anaemia and poverty. Rather than launch a primarily technocratic, health systems strengthening response, the Meghalaya State Capability Enhancement Project (SCEP) focussed on building the capability of government functionaries to implement lasting, decentralised and multisectoral solutions that are adaptive and innovative: solutions locally designed to solve unique local challenges. The Rescue Mission aimed to challenge the default approach of viewing high MMR through a clinical lens alone or assuming that it could not surmount infrastructural and human resource challenges such as limited access to Emergency Obstetric Care Service (EmOC) or ultrasound scanning. Instead, it worked to build a sense of purpose amongst government functionaries and improve coordination between departments, ultimately to encourage problem‑driven, local leadership to address maternal health.

### Methods

As a learning partner to the Government of Meghalaya, Population Council Institute conducted a longitudinal study to examine the implementation of Rescue Mission. The adaptive, embedded nature of the study included regular feedback loops with government leadership and implementers. We drew from implementation research methods [15, 16] to examine intervention content and implementation processes and to identify barriers and enablers, along with contextual factors that influenced implementation outcomes*.* We traced processes at multiple levels (state, district, block, facilities and communities) over a period of 18 months.

**Figure 2:**
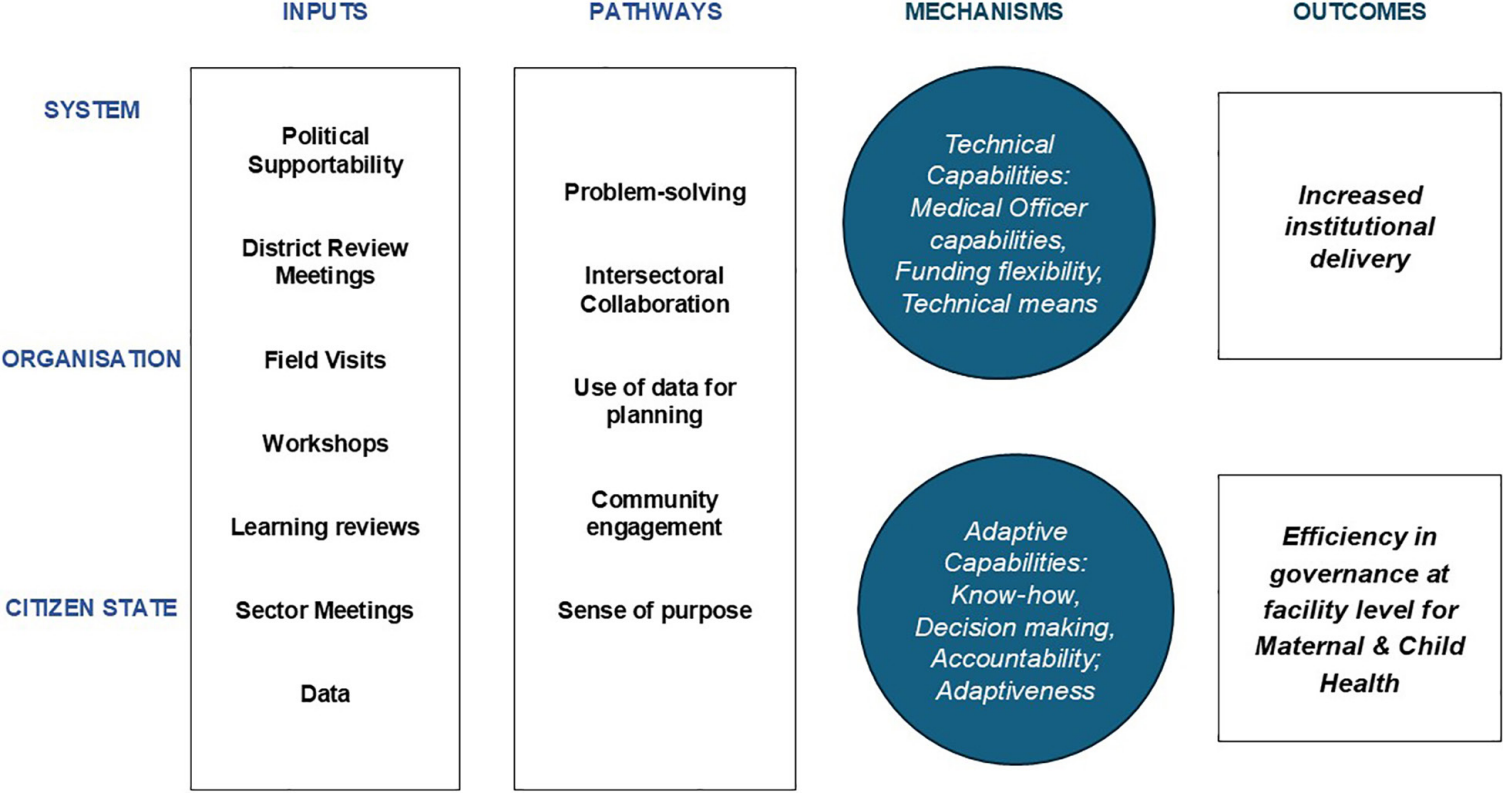
Overarching Theory of Change.

We developed a Theory of Change (ToC) with the Government in two phases. First, we developed a detailed set of implementation frameworks at the community, facility, block, district and state levels to track activities, inputs, processes and implementation outcomes, as well as to consider how interventions interacted together across departments and levels. After observing implementation at each level, we developed an overarching ToC to capture pathways and mechanisms from the view of state capability to address maternal health, which includes key inputs at: the level of the State, Organisation (Health and non‑Health departments) and Citizen–State interaction; pathways of change; and outcomes related to state capability and the health system (Figure 2).

#### Sampling

**Figure 3:**
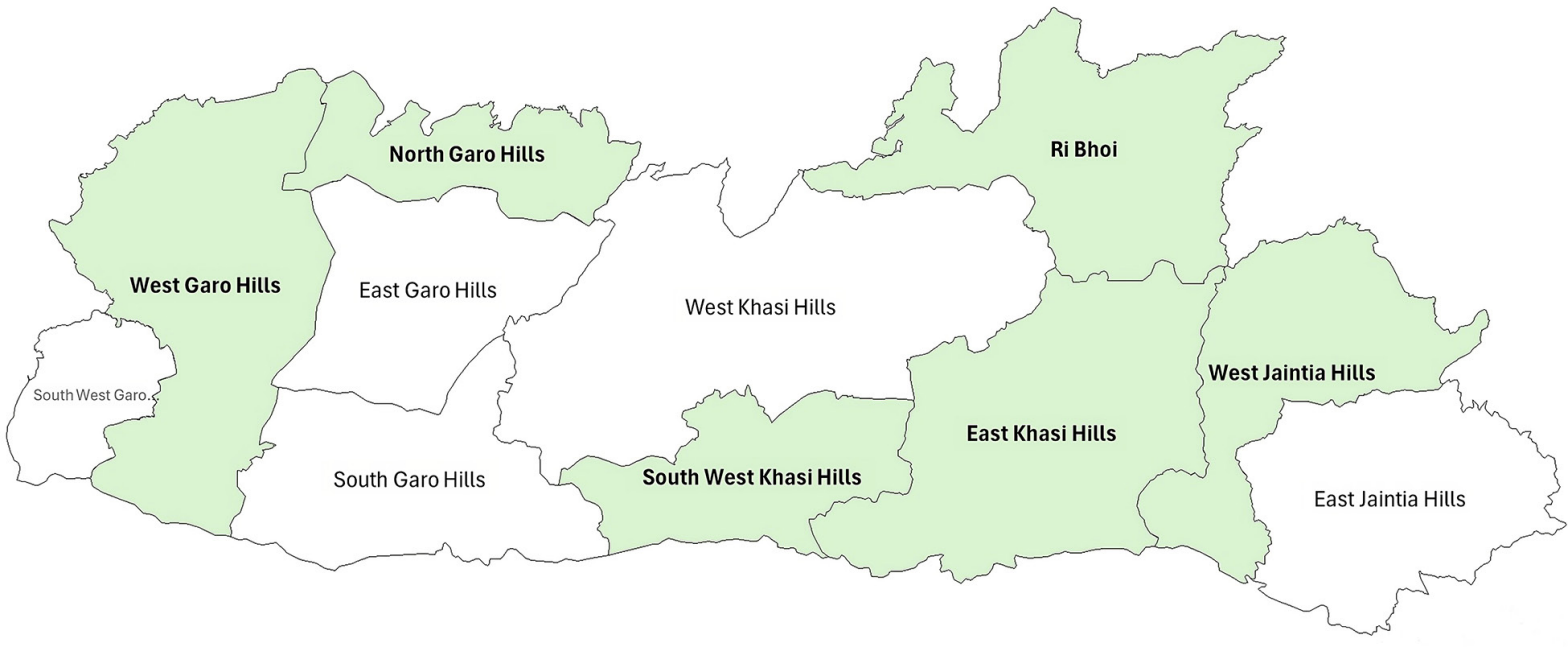
Sampled districts. *Note:* This State map reflects 11 districts; a 12^th^ district, Eastern West Khasi Hills, was carved out of West Khasi Hills just before the study commenced (2021) and was not part of our study*.*

The study’s qualitative process tracking consisted of district, facility and community‑level interviews and meeting observations conducted in multiple rounds in a sample of facilities across the state. We jointly identified six districts to reflect geographic, demographic and health system diversity across Meghalaya: North Garo Hills, West Garo Hills, West Jaintia Hills, Ri Bhoi, East Khasi Hills and South West Khasi Hills (Figure 3). These six districts were chosen purposively to ensure geographic diversity and logistical convenience for repeated interviews. Within these, we selected a sample of 30 Primary Health Centres (PHCs; population coverage <20,000) and Community Health Centres (CHCs; population coverage <80,000) using stratified probability proportional to size based on population at the district level and institutional delivery. Over the course of the evaluation, based on inputs from the Government, we replaced seven sampled facilities that no longer fit study criteria (upgraded to a higher‑level facility or stopped conducting deliveries) or were not implementing Rescue Mission activities.

#### Data sources

Data sources included primary qualitative data, quantitative administrative data from the State’s MOTHER app and secondary information, such as guidelines and relevant reports. Qualitative data included multiple rounds of individual interviews across the different levels of governance, focus group discussions at the community‑level and observations of key Rescue Mission meetings and field visits by the Government of Meghalaya. We tracked monthly administrative data on key indicators (institutional delivery, antenatal care and maternal deaths).

#### Data collection

We conducted interviews with six types of participants at different levels of government over three rounds of data collection. Three investigators, one for each region who could speak respective local languages, focused on facilities. A Khasi‑speaking supervisor conducted interviews at the block and district level. They also conducted focus group discussions with other stakeholders and observed facility‑level meetings, community events and meetings and district‑level review meetings (Table 1). Over time, the team built a rapport with facility staff and block‑level functionaries which enabled repeat interviews as well updates on Rescue Mission activities over time. Regular feedback loops with the government highlighted priorities for learning, based on which we included or excluded stakeholders in subsequent rounds of data collection.

**Table 1:** Data collection.

*IN‑DEPTH INTERVIEWS (IDIs), FOCUS GROUP DISCUSSIONS (FGDs)*					
LEVELS	DEPARTMENT/PARTICIPANT		ROUND I	ROUND II	ROUND III
State	Dept. of Health and Family Welfare, State Rural Livelihoods Society, Dept. of Social Welfare and State Capability Enhancement Project	*IDI*	4		
District	Deputy Commissioner, Medical & Health Officer, Maternal & Child Health Officer, District Community Process Coordinator, District Programme Manager ‑ National Health Mission (DPM ‑ NHM), District Programme Officer ‑ Integrated Child Development Scheme (DPO ‑ ICDS), District Mission Manager ‑ State Rural Livelihoods Mission (DMM ‑ SRLM)	*IDI*	16		
Block	Block Programme Managers ‑ NHM, SRLM, Child Development Project Officer ‑ ICDS	*IDI*	35		
Facility	Medical Officer (MO), MO Ayurveda, Yoga and Naturopathy, Unani, Siddha and Homeopathy (MO AYUSH), Staff Nurse, Auxiliary Nurse Midwife (ANM)	*IDI*	56	43	2
Frontline cadres	Accredited Social Health Activist (ASHA)	*IDI*	27		
	Community Gender & Health Activist (CGHA)				11
Community	Women’s Groups and Village Health Council (VHC)	*FGD*	10		
***Total IDIs and FGDs***		***194 IDIs, 10 FGDs***			
*Note: While number of participants are distinctly reported, data collection was continuous between rounds II & III.*					
**Observations**			**November 2022 – November 2023**		
All District and State Review Meetings*			27		
All Sector Meetings*			66		
State Workshops			2		
Field visits by Government leadership			5		
Field observation of health providers			5		
VHC meetings			1		
***Total observations***			***106***		

#### Ethical considerations

Ethical approval was granted by the Sigma Institutional Review Board (Reference No. 10068/IRB/22‑23, 5/11/2022). Trained investigators administered informed consent procedures and recorded audio only after permission from participants.

#### Analysis

After every round of data collection, recordings were translated and transcribed from Khasi, Garo and Pnar into English, the team’s common language, and prepared for coding and analysis. The research team held internal workshops to code and analyse data thematically. Observation notes by field investigators supplemented transcripts. We removed identifying information for respondents and used pseudonyms in this manuscript. Themes that emerged across three rounds of data collection included sense of purpose, decentralised and adaptive leadership, political supportability, ownership, health system factors, accountability mechanisms, community engagement and use of data. Themes were shared with the Government to understand priorities for subsequent rounds of data collection and to validate interim findings.

We developed an index of state capability approach adoption and categorised Medical Officers (MOs) (based on interviews) across five categories: problem‑solving approach; intersectoral collaboration; use of data for planning; community engagement and sense of purpose. We used a 3‑point scale (limited, average and exceptional) for scores between 5‑15. The intention of mapping facilities by these indices was to identify opportunities to strengthen inputs rather than to rank MOs on performance.

## Findings

We present trends in maternal health outcomes, followed by qualitative findings aligned with the Rescue Mission’s key pathways: multisectoral collaboration, decentralised leadership and community mobilisation, with specific sub‑themes.

### Maternal health outcomes

**Figure 4:**
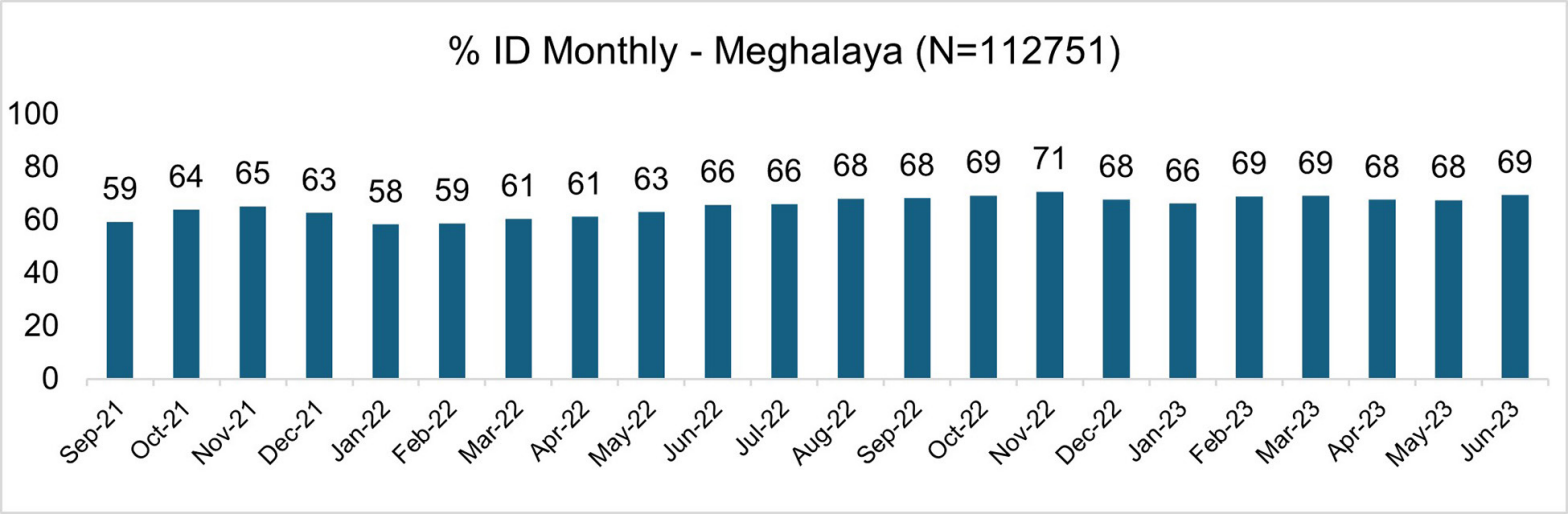
Monthly Institutional Delivery Rate; September 2021‑Jun 2023 (% of deliveries). Source: Mother app data, provided by Govt of Meghalaya.

The institutional delivery rates steadily increased from 59% in September 2021 to 69% in June 2023 (Figure 4). The Garo region had an institutional delivery rate of 67%, similar to the Jaintia and Khasi regions (65%). Maternal deaths reported in the State registry reduced from 204 (from September 2021‑2022) to 121 during September 2022–2023 [17].

### Multisectoral collaboration

Multisectoral collaboration between the Departments of Health & Family Welfare, Community & Rural Development and Social Welfare is institutionalised across governance levels and through community outreach activities. Specifically, Rescue Mission engages the Department of Social Welfare’s Integrated Child Development Scheme (ICDS), an early childhood development programme which aims to address health and nutrition needs of young children, pregnant and nursing mothers and the State Rural Livelihoods Mission (SRLM) that organises women’s Self‑Help Groups (SHGs). Officials in both departments reflected a commitment to improving maternal and child health. Amongst officials of the SRLM, there was a recognition of the clear link between livelihoods and social determinants of maternal health, while the Social Welfare department viewed their role as primarily focused on reducing anaemia and improving nutrition.

*“One thing that the Rescue Mission has taught us is that all interventions are interlinked.”*

– District official, SRLM

*“If they do not work together then the whole Rescue Mission will be of no use”*

– District official, ICDS

### District, sector and facility‑level review meetings

Meetings across sectors were the central method of fostering collaboration. At the State‑ and district‑level, meetings first articulated responsibilities by department, reviewed progress on key health priorities, identified implementation and coordination challenges and developed tailored solutions. After a round of covering all districts, an all‑District Review Meeting was convened by State leadership, chaired by the leadership of any of the three departments. The Health Department typically steered these more often than other departments. District officials viewed these meetings as a platform for cross‑district learning as well as problem‑solving for identified challenges. We observed that meetings helped sustain a sense of purpose and motivation across departments; target‑setting and evaluation by each District/State Department promoted a sense of urgency.

“*The review meetings remind us to do those activities and to finish the work we have.*”

– District official, SRLM

“*Without meeting it will be chaos. … The meetings are useful because without them they won’t be able to go further. They won’t know what to do.*”

– District official, NHM

At the facility level, reviews (Sector meetings) were intended to facilitate teamwork across the three departments, including facility‑level and frontline cadres updates and review of any maternal or child deaths. If any challenges were identified, the Sector Team would attempt to find solutions during the meeting. These meetings also offered a platform to raise facility‑level and department‑specific issues. In our observation, Sector Meeting action plans tended to be generic. For instance, an MO would direct ANMs to follow up on dropouts for immunization without specifying how many were to be followed up and specifically in what communities. In exceptional cases, facilities developed concrete action plans with accountability for specific items.

The regularity of meetings appeared to vary by facility, often due to scheduling conflicts between the three departments. As a result, some districts mandated specific days in the month for Sector meetings. Other districts further mandated meetings be held in villages with particularly poor health indicators such as immunisation and institutional deliveries. Many facilities combined Sector meetings with other scheduled meetings at the Block‑level. The strength of participation of all departments varied across geographies. Functionaries from non‑Health departments recognised the value of Sector meetings but found it challenging to attend them regularly. Despite variation across meetings, there appeared to be a consistent, and perceptible, increase in collaboration due to joint planning, review and monitoring during meetings.

*“If there is no meeting then the three line departments would not have come together and worked. Because of this meeting we are getting the opportunity and we are utilising it so we can see improvements.”*

*–* Health Provider

“*Sector meetings were started with good intentions, but I don’t feel that it is serving its purpose. Other line departments are not as serious and see the responsibility for maternal and child health to be that of the Health Department*.”

– Medical Officer

### Transit homes

Transit homes located within or near health facilities for high‑risk pregnant women were introduced to facilitate timely access to delivery care for women with poor road connectivity, managed by SHGs and local entrepreneurs (Box 1) [18]. Uptake varied across geographies. Women who had previously given birth at home found it challenging to leave their children and household responsibilities to stay away for a prolonged period. Women who used Transit Homes were largely from areas that had non‑motorable roads. In Blocks with hard‑to‑reach areas but motorable roads, families preferred to rely on vehicles than stay in transit homes.

Nearly 94 transit homes were being run by SHGs; stakeholders perceived a clear difference in transit homes run by women’s groups because they were able to better support and engage pregnant women. Health facility staff in contrast were unable to engage with them in a similar manner. Some invited traditional birth attendants – trusted by the community – to run transit homes as a potential solution.

*“In that Transit home, all the SHG members came and sat down with the mother and started to talk. Spending time, a support system was there. When they left, somebody else came and brought food so that the mother during that period when she was staying at the Transit home she said she did not feel lonely at all.”*

– State official

**Box 1. Chief Minister’s Safe Motherhood Scheme (CM‑SMS)**

In 2022, the Government of Meghalaya announced the Chief Minister’s Safe Motherhood Scheme (CM‑SMS) with the goal to improve maternal healthcare in the state. The Scheme consists of five key components: a) mobility support to health facility staff to undertake community outreach visits; b) dedicated maternity care vehicles at facilities for use by pregnant women; c) set up of transit homes (maternity waiting homes) in or next to facilities; d) compensation for loss of livelihoods to attendants or traditional birth attendants accompanying pregnant women to facilities; and e) rewards to Village Health Councils for achieving targets.

### Decentralised leadership

Most Rescue Mission activities are centred on the leadership of Medical Officers (MOs) at Primary Health Centres, who in turn collaborate with facility and block‑level teams in local problem‑solving.

### Sense of purpose

A central goal of SCEP was to build a sense of purpose and motivation amongst frontline workers and providers, ultimately to transform public service delivery through decentralised, local leadership with the freedom to make decisions. Accordingly, Rescue Mission cultivated Medical Officers (MOs) as leaders of their facilities and surrounding areas, which would also free District level leaders to focus on broader monitoring and support.

“*Strengthening MOs, giving them* authority*, again and again talking to them… otherwise the government only functions at the top level*.”

– State official

Amongst interviewed MOs, nearly all appeared to draw their sense of purpose from serving their communities. They described experiencing deep satisfaction from being able to treat patients who had travelled great distances to access care with limited resources. Positive feedback from patients and the community’s trust in the MOs were highlighted as sources of encouragement.

*“I want to be close to the community. I heard about the PHC from a friend. This means living with and helping the community. I decided to go and work there to see if it suits me. I want to be with patients and I am actually happier here.”*

– MO

*“It is the trust of the people towards me that motivates me to do my work from day to day.”*

– MO

MOs also reported sources of motivation that went beyond service provision, such as feeling invested in the overall development of their communities, improved performance in schools in their area or increased male participation for health through Village Health Councils.

*“Job satisfaction is when me and my staff, working tirelessly to save lives of patients and pregnant woman, provide them with exceptional care. It is during these moments that I find fulfilment in my job.”*

– MO

On the other hand, several MOs we interviewed reported inadequate human resources as a primary source of demotivation. They reported feeling overworked, finding it difficult to give all their patients and programmes sufficient time. They did however recognise that their work required them to be available 24 hours a day, while engaging in non‑clinical work such as facility administration and monitoring. MOs also reported difficult living and working conditions, particularly poor facility infrastructure, electricity and water. While these issues were perceived to affect service provision, they recognised that these extended beyond the purview of the Health Department.

Some MOs felt demotivated to perform their work when they observed low strength of participation from non‑health departments, overall slow government processes and inadequate compensation. They highlighted requiring support and capacity strengthening to handle some of the managerial, financial, and administrative aspects of their jobs for which they felt unprepared.

*“But I love working here. The only thing I feel sad about is the infrastructure. The services we give are not 100%.”*

– MO

*“We are never taught how to administer a hospital or manage its operations, which burdens us. Additionally, we lack knowledge of financial management for Primary Health Care (PHC) and yet are compelled to handle it. Furthermore, we face a high workload with limited manpower. Despite these challenges, the remaining aspects are positive.”*

– MO

*“As a Medical Officer, I feel demotivated because I am unable to dedicate enough time to my patients and unable to provide them quality care due to the programs after programs and reports, even during late hours, leaves me with no peace of mind and no proper rest.”*

– MO

### Problem‑solving

MOs appeared to take independent decisions on task delegation, day‑to‑day functioning of the facility as well as minor infrastructure‑related issues. For larger purchases and actions, MOs typically deferred to the Block‑ or District‑level decision‑makers. Most MOs perceived not having the required finances or human resources to implement actions for tackling bigger issues. However, they did appreciate the flexibility in spending that the Chief Minister’s Safe Motherhood Scheme allowed; MOs devised innovative, context‑specific solutions to transportation through this flexibility (Box 2). Facility visits by State officials appeared to serve as a direct line of communication between MOs and the State. Visits by technical health consultants allowed for direct grievance redressal, whereas MOs were more reluctant to share problems with State‑ and District‑level authorities during Review meetings.

**Box 2. Local Problem‑Solving**

Over the course of data collection, we found several instances of innovative problem‑solving by MOs and their staff. In some facilities, MOs clearly delineated work responsibilities amongst staff members to ensure that facility‑based care and community outreach work were evenly distributed. In an area with poor network connectivity, ANMs were unable to communicate with ASHAs to inform them of focused outreach visits. Unaware, community members would then miss out on crucial services such as immunisation camps. Towards the end of the study, we found that ASHAs and ANMs had overcome this difficulty by fixing specific dates in advance. On a related note, ANMs found it difficult to commute in the absence of public transportation. The MO hired bikes instead of cars as poor road conditions didn’t support travel by cars / shared taxis. In another area with non‑motorable roads, an MO identified specific individuals to carry pregnant women to the facility in locally crafted chairs.

Many facilities have found ways to work with trusted Traditional Birth Attendants instead of isolating them. Some MOs have invited them to run their transit homes, thus providing pregnant women someone with whom they are comfortable. Others encouraged them to bring pregnant women to the facility and have trained them in identifying early danger signs.

### Use of data

The State government launched the MOTHER app in 2019 to improve monitoring of data on maternal and child health. The app is meant to integrate real‑time data from the field collected and inputted by frontline staff (ANMs, ASHAs) for doctors and health officials at the block‑, district‑ and state‑levels to track progress through a dashboard. ASHAs and ANMs reported that the MOTHER app was one amongst many apps and portals across health programmes that they were expected to update, resulting in a time‑consuming process. Lack of network connectivity was identified as a key barrier to timely uploading of data. Capacity to use these apps appeared mixed, with older ANMs and ASHAs facing difficulties. Manual record‑keeping appeared to supplement data recording on apps. Data pertaining to the Rescue Mission was also shared with functionaries in the Social Welfare and Rural Development departments at the Block and District levels. The district‑level leadership used data to identify problems, such as tracking trends, identifying problem areas and informing action plans.

*“For us all, these activities have become regular.”*

– District official, NHM

MOs drew on data to monitor performance on key service indicators during review meetings, but the degree of analysis and depth of action plans varied. Some MOs reported challenges such as requests for reporting in multiple formats with tight deadlines and poor network connectivity, as well as not being aware about how Blocks and Districts utilised the data they reported.

### Adopting the state capability enhancement approach

**Figure 5:**
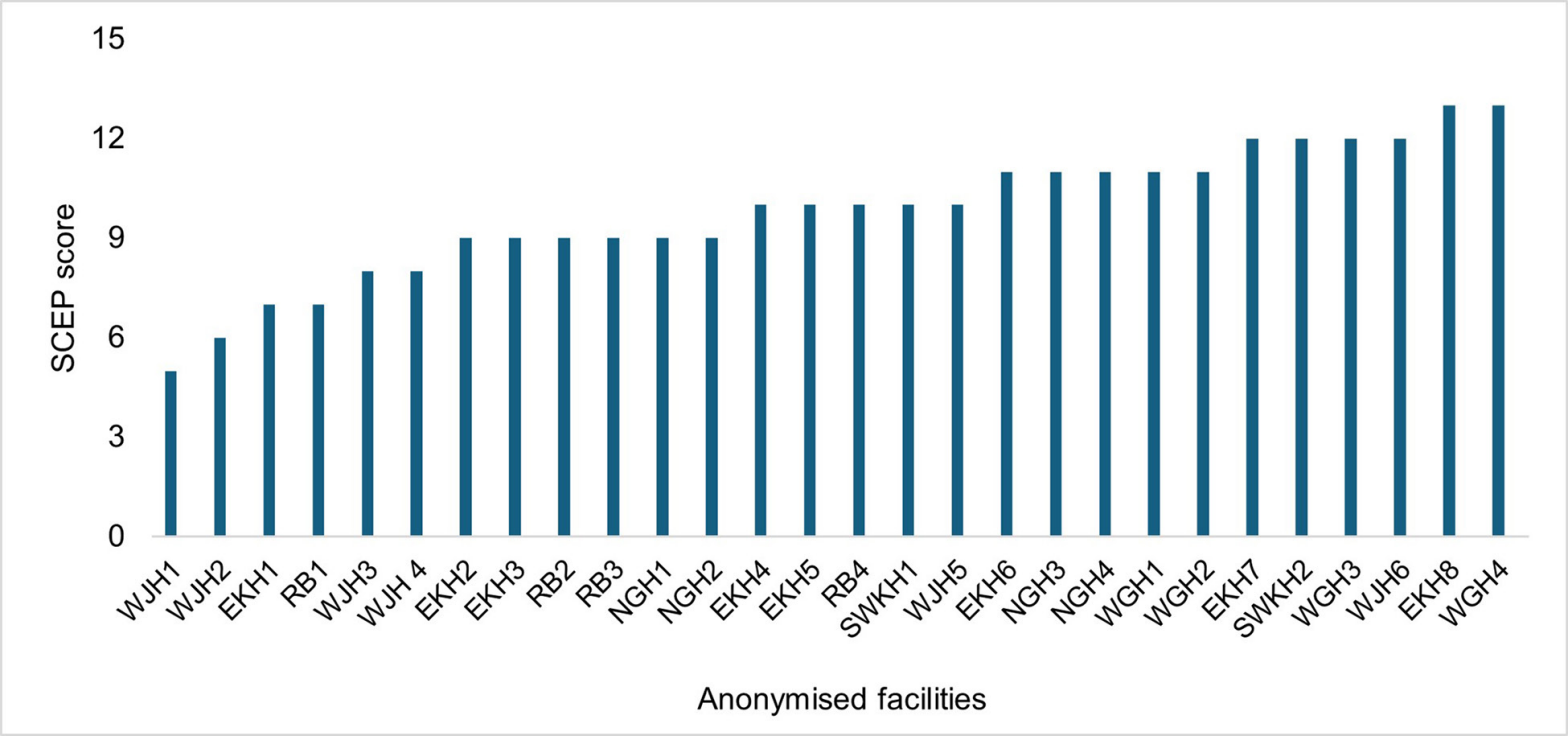
MO adoption of the State Capability Enhancement approach.

MO adoption of SCEP principles ranged from a score of 5 to 13, with a median score of 10 (Figure 5). The index suggested that most MOs adopted the basic principles, but did not go beyond in most cases. Most importantly, we noted the variation in MO approach did not correlate with the type of PHC, system performance (such as institutional delivery rate) or context. Rather, sense of purpose and problem‑solving capabilities emerged as intrinsic qualities.

### Community mobilisation

The Rescue Mission implemented community mobilisation through the combined, multisectoral strength of frontline workers (SHGs, ASHAs and Anganwadi workers). SRLM officials recognised that the strong social capital of SHGs was a valuable contribution to improving maternal health. However, some noted diversion from their main activities in a context with human resource shortages and increased demands for data reporting. The Department of Social Welfare had already collaborated with Health on overlapping population groups of pregnant women and children, such as health check‑up camps.

“*Earlier when people from the health department used to go to the village, we would not get a response from the community. But now when we go along with the other departments who provide schemes for the rural development, the people in the community started to respond to us.”*

– NHM

*“Now, when they conduct home visits they have a specific objective in mind, whereas earlier, they would conduct home visits for the sake of completing their work.”*

– Block official, ICDS

The Rescue Mission introduced a new cadre of Community Gender & Health Activists (CGHAs) drawn from SHGs. With their different backgrounds, CGHAs were to focus on social determinants such as food, nutrition and sanitation to support the ongoing work of ASHAs. They capitalised on a closer rapport with community members, as they were drawn from SHGs, compared to ASHAs who were seen as functionaries of the Health Department. At the time of data collection, grassroots convergence appeared to be strong only in geographies where SHGs were active and already interacted with the heath system. CGHAs highlighted certain challenges to their work, such as long working hours, a lack of transportation to conduct field visits and walking long distances in hard‑to‑reach areas. Given the overlapping mandate between the two departments, CGHAs noted difficulties with coordination as they found themselves answerable to functionaries across two departments.

### Village health councils

Village Health Councils (VHCs) were introduced in 2022 as community institutions to build community ownership for health and nutrition in Meghalaya. Meant to serve as a link between the health system and communities, they have replaced previous Village Health, Sanitation and Nutrition Committees (VHSNCs). VHCs comprise of elected rather than appointed members, including leadership from Meghalaya’s traditional institutions and village organization presidents from women’s SHGs.

At the time of data collection, VHCs were just being set up and therefore, relatively new. However, we noted that in communities where VHSNCs were active previously, VHCs continued similar activities and demonstrated community ownership. Some VHCs activated non‑functional Sub Health Centres and headmen emerged as active leaders who built on previous experience creating community‑owned assets through Village Employment Councils.

*“There is tremendous credibility for traditional institutions. They have never been given the responsibility. When we gave responsibility during COVID‑19 times they did very well.*”

– State Official

### Reflections with government

The theory of change posited changes in both technical and adaptive capacity of the health system and specifically MOs. While technical capabilities in using data or implementing programmes could be strengthened directly through training, the adaptive capability of MOs emerged as intrinsic skills that were not built through external inputs. At the system level, a deepened ownership of intersectoral collaboration – a longstanding challenge in governance – emerged as a key achievement of continued efforts through joint meetings and collaboration at the front line. Discussions with government after our first round of findings led to recognition of the need for closer engagement with MOs directly through local sessions, tailored training and learning across districts—rather than relying on Review Meetings to strengthen problem‑solving capability. The government leadership articulated that the central challenge for the Rescue Mission was to intensify efforts at building the “soft architecture” to achieve success in implementation, rather than technocratic approaches to problem solving.

## Discussion

The Government of Meghalaya applied a state capabilities approach to maternal health, and in the process shifted the focus of a health systems strengthening programme to building leadership. The ToC assumed that Rescue Mission’s efforts to build technical and adaptive capabilities would ultimately support improvements in institutional delivery and more efficient governance. At the population level, institutional delivery has clearly improved over time according to administrative data, along with a distinct downward trend in maternal deaths, which will be further confirmed through the next round of the National Family Health Survey. SCEP intensified focus on building a sense of purpose and commitment rather than technical capabilities alone, with a specific aim to mobilise frontline workers to optimise resources to save lives. The goal of Rescue Mission was to provide greater autonomy and a sense of leadership, with the assumption that this would generate intrinsic motivation.

In Meghalaya, clearly established processes for multisectoral collaboration and the use of data at local levels indicate the effectiveness of Rescue Mission in strengthening technical capabilities. Institutionalised meetings across departments enabled a multisectoral approach, but the frequency, regularity and strength of participation varied widely. Another enabler to reaching the most vulnerable, particularly women, was community engagement through a multisectoral approach that engaged frontline workers across three departments. These findings resonate with recent global evidence syntheses on multisectoral interventions to improve health, particularly their critical role in community engagement and in breaking silos [8, 19].

Further, the concept of Rescue Mission, as well as the salience of maternal health as a priority, has spread across levels. Decentralisation efforts focussed on leadership and financing, which insights from several settings indicate are essential components to support overall health system strengthening [20]. Decentralisation gradually permeated through processes as well; Village Health Councils, though nascent, represent a potential path towards localised financing and priority‑setting and, by extension, accountability [21].

The primary challenge, however, was that building a sense of purpose required more inputs beyond Rescue Mission. Our findings suggested that motivation appeared to be intrinsic amongst some MOs, with no clear pattern of demographic characteristics or regular exposure to review meetings. Faced with these learnings, the Government has introduced a new effort to inculcate leadership skills. The Human Development Leadership Programme facilitates regular engagement with, and exposure to, the lives of the poorest through visiting every household, to understand their problems and build trust in government [22]. Rather than build only technical skills, the Government recognised that decentralised leadership to serve the most vulnerable requires building “capability through empathy.”

### Transferability

Three elements of Rescue Mission offer lessons for other settings on multisectoral approaches towards health. One, political leadership was a central, non‑negotiable feature – which overcame a well‑known challenge that the “most important barriers to multisectoral action are political rather than technical” [23]. Political support for maternal health was actively nurtured by policymakers from the outset, exemplified by the widely promoted Chief Minister’s Safe Motherhood Scheme. Maternal health could attain political priority in Meghalaya because it represented a convergence of problems and policies at the national and state‑level, further supported by an international focus on MMR in the SDGs [21, 24, 25]. The signal sent from political leadership established that maternal health was “everyone’s problem” and not solely that of the health department. Two, administrative leadership demonstrated problem‑solving in practice, both during State‑level meetings and facility visits. The importance of seeing empathetic leadership in practice emerged in reflections from interviewees, particularly the power of personal visits to facilities and following through on commitments of hands‑on support.

Three, building the case for maternal health for non‑health departments contributed to ownership. This process required communicating clear linkages or finding a common area of interest between health and mandates which fell outside the purview of health but addressed social determinants of health nonetheless. The department of Social Welfare had a natural overlap with activities on early childhood development and nutrition. However, with the Rural Development department, percolation of linkages between good health and improved livelihoods – a key aim of their work – from the State to grassroots levels required time and effort, especially for frontline cadres who had increased work as a result of multisectoral efforts. To build consensus for multisectoral action for health, clarity of linkages is essential for the buy‑in of non‑health departments, which was relatively straightforward for maternal health but may require further efforts for different health issues [23, 26].

Conceptually, the government’s focus on adaptive, rather than only technical, expertise drew from the problem‑driven iterative approach (PDIA), which supports governance methods that promote: local solutions for local problems; problem‑driven positive deviance; active learning and experimentation; and scaling through diffusion through engaging champions [27]. While PDIA specific to health systems and governance is an emergent field, its principles—albeit with a different set of actors—resonate with a well‑established method in India to address maternal health through engaging women and mobilising communities through cycles of participatory learning and action [28]. The further evolution of SCEP in Meghalaya to building empathy through active, grassroots engagement echoes wider experience in India with early government‑promoted self‑help groups in Andhra Pradesh that engaged in extensive, bottom‑up village‑level mobilisation [29].

### Strengths and limitations

The embedded nature of the study allowed for “insider” access to review meetings, audits and most importantly, a rapport with leadership to discuss findings and shift focus as needed. To our knowledge, this study was amongst the first to examine application of a state capability approach to the health system; future efforts can expand methodological approaches accordingly. The exploratory, adaptive nature of this study allowed us to intensify focus on MO motivation mid‑way, for example. Future research can build on this with more structured evaluation of the impact of the Human Development Leadership programme and to identify mechanisms that influence building purpose [8, 30]. Regular interviews and visits to facilities allowed for in‑depth insights from a sample of MOs, although supplementation with a quantitative survey across the state may have facilitated greater insight into variation across the state. Moreover, future research should integrate more extensive research with community institutions and service users to supplement the perspectives of providers and government officials.

## Conclusion

The Rescue Mission offers new insights into how an Indian state institutionalised multisectoral action to improve maternal health. Most importantly, it demonstrates how to implement participatory, people‑driven processes at both the systems and community level to improve to health systems governance. To facilitate application to other health domains and to scale up to other settings, this combined state capability and health systems strengthening approach calls for strong political and administrative leadership, building ownership through a continuous progress of reflective program implementation and importantly, a commitment to enhancing local, empathetic leadership to reach the most vulnerable.
